# Correction: Khan et al. Anticancer Function and ROS-Mediated Multi-Targeting Anticancer Mechanisms of Copper (II) 2-hydroxy-1-naphthaldehyde Complexes. *Molecules* 2019, *24*, 2544

**DOI:** 10.3390/molecules26196070

**Published:** 2021-10-08

**Authors:** Muhammad Hamid Khan, Meiling Cai, Jungang Deng, Ping Yu, Hong Liang, Feng Yang

**Affiliations:** State Key Laboratory for the Chemistry and Molecular Engineering of Medicinal Resources, Guangxi Normal University, Guilin 541004, China; hamidgxnu@hotmail.com (M.H.K.); jiangbiochem@163.com (M.C.); zhangzl@mailbox.gxnu.edu.cn (J.D.); zzlei18@163.com (P.Y.)

Due to the previous incorrect characterization of compound **C1**, the authors wish to make the following corrections to this paper [1] published in Molecules.

In order to be easier for the reader to follow, a short text from the original manuscript is cited together with the respective modification.

In Section 2.1. Development and Structure of Cu^2+^ Complexes:

Original:

**C1** was crystallized in the space group P21/n making a monoclinic structure. The molecular structure of the arrangement of the Cu (II) metal center is listed in Table 1. The Cu (II) metal center was pentacoordinate with two nitrogen atoms and an oxygen atom of the ligand and two terminal chlorine atoms. The distances of the Cu–N/O bonds ranged from 1913–2.02 Å (Table S1). The coordination of the polyhedron surrounding the Cu atom at the center could be displayed by a square pyramid, with the metal displaced from the N1/N2/O basal plane, ranging from 88–175.9° (Table S2).

This should be replaced with:

**C1** was crystallized in the space group P21/n, making a monoclinic structure. The molecular structure of the arrangement of the Cu (II) metal center is listed in Table 1. The Cu (II) metal center was pentacoordinate, with two nitrogen atoms and an oxygen atom of the ligand, and one terminal chlorine atom. The distances of the Cu–N/O bonds ranged from 1.895 to 2.009 Å (Table S1). The coordination of the polyhedron surrounding the Cu atom at the center could be displayed by a square planar, with the metal displaced from the N1/N2/O basal plane, ranging from 82 to 176.7° (Table S2).

In Section 4.2.2. Synthesis of **C1**:

Original:

L and CuCl_2_ (0.5 mmol) were stirred for 1 h at 65 °C to give a clear solution and then filtered. The filtrate was kept in the air for a week, forming blue-black crystals. The crystals were directly isolated, washed three times with distilled water, and dried in a vacuum desiccator containing anhydrous CaCl_2_. Yield: 83%. Anal. Calc for C_17_H_13_Cl_2_CuN_2_O (395.75): C, 51.59; H, 3.31 and N, 8.68. Found: C, 51.51; H, 3.24 and N, 8.60. IR (Main Peak cm^−1^): 3461.33, 3090.05, 1719, 1601.92, 1344.26, 761.53, 702.21. MS m/z: 396.75 (M + H)^+^ (Tables S1 and S2).

This should be replaced with:

L and CuCl_2_ (0.5 mmol) were stirred for 1 h at 65 °C to give a clear solution and then filtered. The filtrate was kept in the air for a week, forming blue-black crystals. The crystals were directly isolated, washed three times with distilled water, and dried in a vacuum desiccator containing anhydrous CaCl_2_. Yield: 83%. Anal. Calc for C_17_H_13_ClCuN_2_O (324.03): C, 56.67; H, 3.64 and N, 7.78. Found: C, 56.61; H, 3.70 and N, 7.72. IR (Main Peak cm^−1^): 3461.33, 3090.05, 1719, 1601.92, 1344.26, 761.53, 702.21. MS m/z: 324.03 (M − Cl)^+^ (Tables S1 and S2).

The Figure 1 should be replaced with the following version:

The Table 1 should be replaced with the following version:

**Table 1:** Crystal data of the Cu (II) complexes (**C1** and **C2**).

Identification Code	C1	C2
Empirical formula	C_17_H_13_ClCuN_2_O	C_22_H_18_BrCuN_3_O
Formula weight	360.30	483.86
Temperature/K	296.15	296.15
Crystal system	monoclinic	monoclinic
Space group	P21/n	P21/n
a/Å	8.100(6)	8.6992(9)
b/Å	8.632(7)	13.6084(14)
c/Å	21.270(16)	16.4250(18)
α/°	90	90
β/°	100.245(12)	90.070(2)
γ/°	90	90
Volume/Å3	1463.4(19)	1944.4(4)
Z	4	4
ρcalcg/cm^3^	1.6353	1.6527
μ(Mo-Kα) mm^−1^	1.677	3.196
F(000)	734.2	972.6
Data/restraints/parameters	3007/0/199	4211/0/253
Goodness of fit on F2	1.054	1.027
Final R indexes (I ≥ 2σ (I))	R1 = 0.0421	R1 = 0.0375
	wR2 = 0.1358	wR2 = 0.1023
Largest diff. peak/hole /e Å-3	0.63/−0.69	0.86/−0.46

All co-authors agree with the content of this correction and wish to apologize for any inconvenience to the readers resulting from this error.

## Figures and Tables

**Figure 1:**
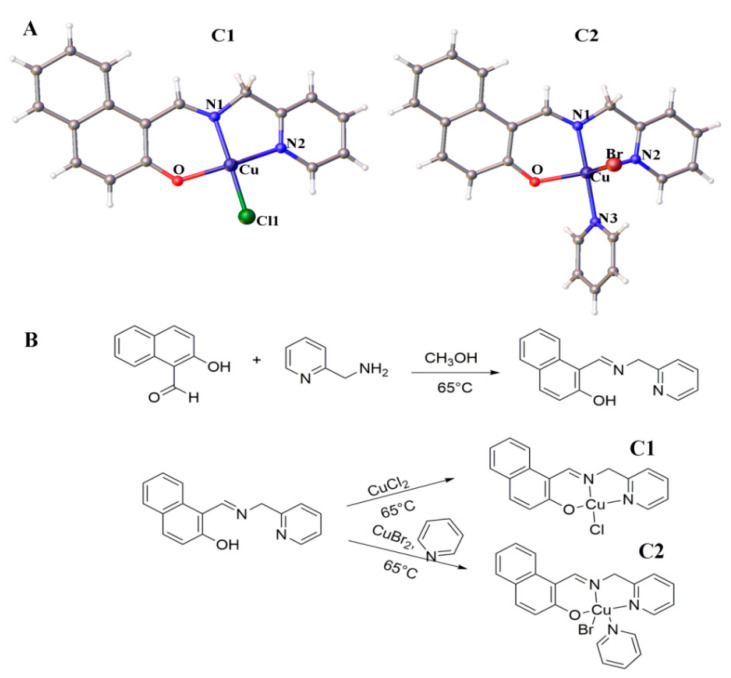
(**A**) Chemical structures of Cu^2+^ compounds. (**B**) Synthetic routes of Cu^2+^ compounds (**C1** and **C2**).
