# Supplementary material for: Anticancer Function and ROS-Mediated Multi-Targeting Anticancer Mechanisms of Copper (II) 2-hydroxy-1-naphthaldehyde Complexes
Source: Molecules. 2019 Jul 12;24(14):2544. doi: 10.3390/molecules24142544 (PMC6680819; doi:10.3390/molecules24142544)
Supplement: Supplementary file 1 [file molecules-24-02544-s001.pdf]

Supporting Information

ROS-mediated multi-targeting anticancer mechanisms of copper (II) 2-hydroxy-1-naphthaldehyde complexes

Muhammad Hamid Khan, Meiling Cai, Jungang Deng, Ping Yu, Hong Liang*, Feng Yang*

Table of Contents

1. X-ray crystallography C1 and C2
2. bond length [Å] in complexes C1 and C2
3. 3bond angles [°] in complexes C1 and C2 2
4. UV-Vis spectra of the complexes' stability (C1, C2)
5. ESI-mass spectrum of the Ligand
6. ESI-mass spectrum of the C1 5
7. ESI-mass spectrum of the C2 6
8. IR spectrum of Ligand
9. IR spectrum of C1 7
10. IR spectrum of C2

X-ray crystallography C1 and C2

Single crystals of C1 and C2 were selected and analyzed by Burker APEX-II CCD diffractometer. The crystals were kept at 296.15K during data collection. The structures were solved by using Olex2. The crystals data of two complexes are listed in tableS1. The selected bond lengths (Å) and bond angles (°) of crystal structure are listed in Table S1, S2.

Table S1. Selected bond length [Å] in complexes C1 and C2.

Identification Cod	C1 Bond Length(Å)	C2 Bond Length(Å)
Cu-1—O-1	1.913	1.917
Cu-1—N-1	1.965	1.973
Cu-1—N-2	2.02	2.02
Cu-1—N-3	----	1.926
Br-1—Cu-2	----	2.8458
Cu-1—Cl-2	1.998	----

Table S2. Selected bond angles [°] in complexes C1 and C2.

C1		C2	
**Identification Code**	**Bond Angles (°)**	**Identification Code**	**Bond Angles (°)**
O—Cu—Cl-1	88	O—Cu—Br	108.22
O—Cu—N-2	91.8	N-2—Cu—Br	95.61
O—Cu—N-1	155.3	N-2—Cu—O	87.41
N-3—Cu—Cl-2	175.9	N3—Cu—Br	88.33
N-3—Cu—N-1	81.8	N3—Cu—O	91.61
N-3—Cu—Cl-1	96.7	N3—Cu-2—N-2	176.06
C-2—O—Cu	128.9	N-1—Cu—Br	93.23
C-3—N-3—Cu	122.7	N-1—Cu—O	157.62

C-4—N-1—Cu	112.8	N-1—Cu—N-3	82.48
C-7—N-3—Cu	115.6	C-2—O—Cu	127.5
C-6—N-1—Cu	126.2	C-7—N-2—Cu	120.1

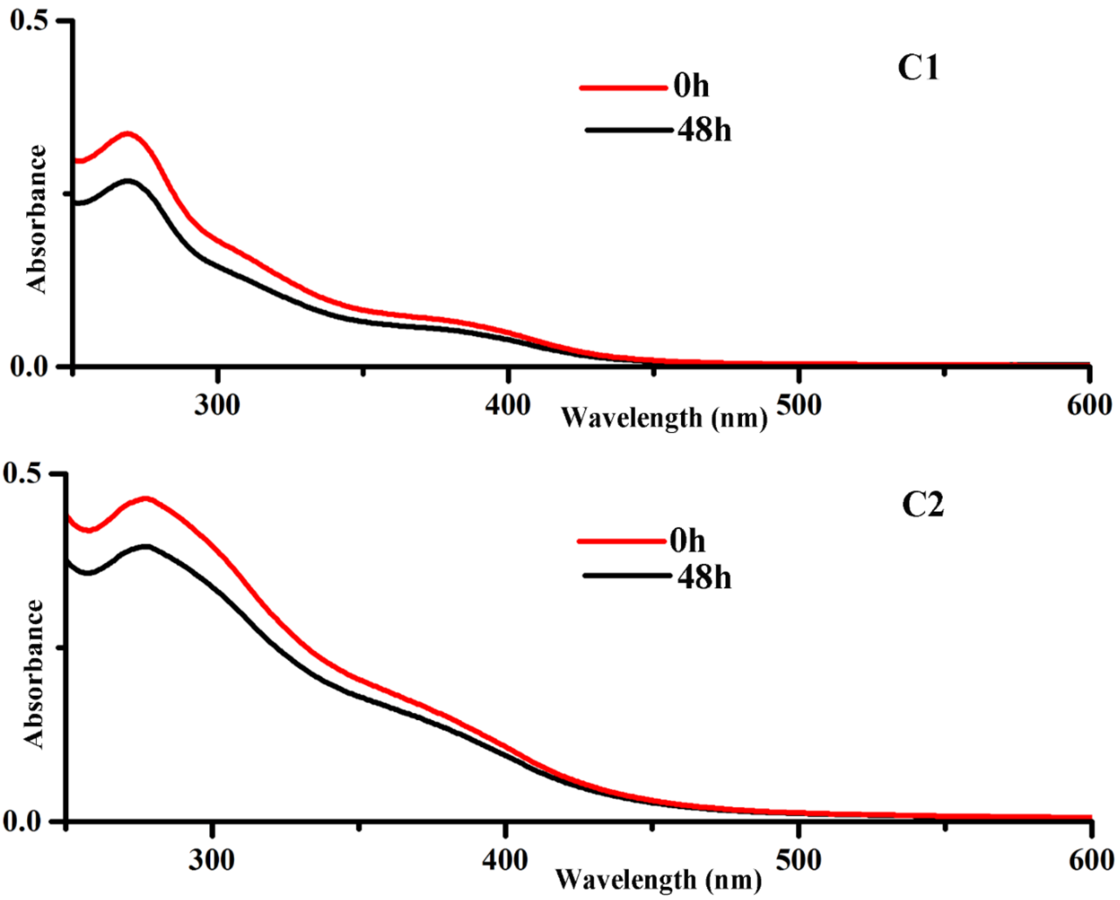

Figure S1. UV-Vis spectra of the complexes' stability (C1, C2).

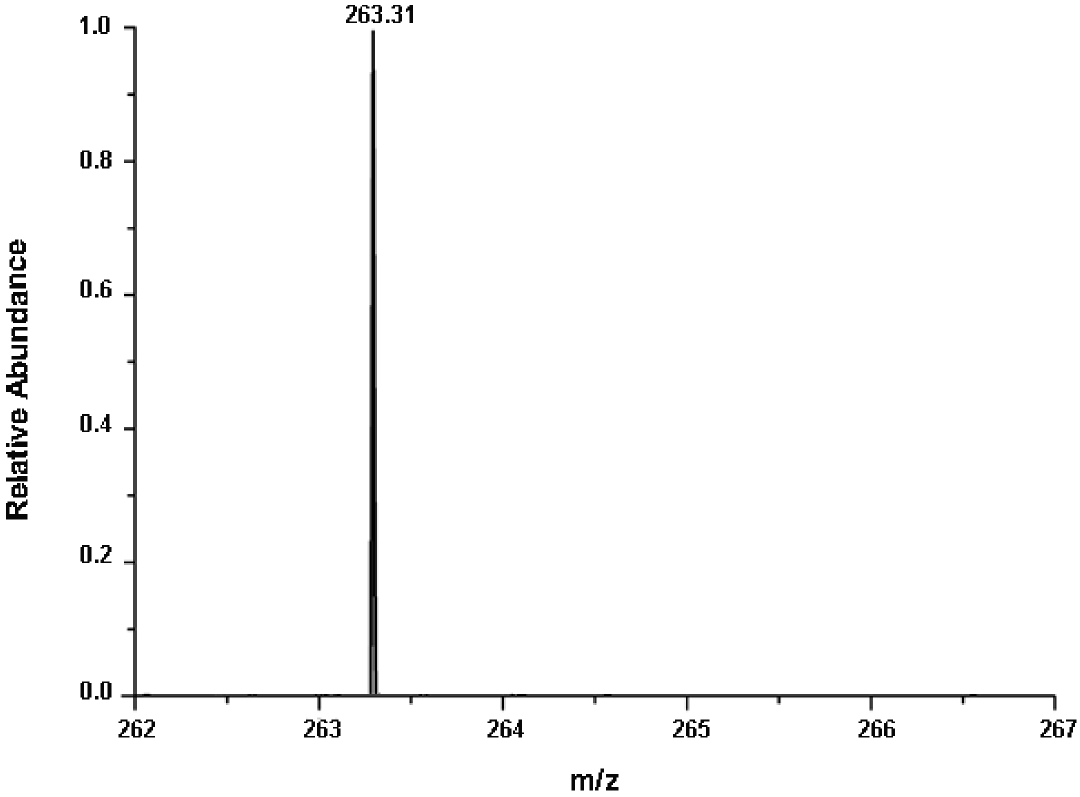

Figure S2. ESI-mass spectrum of the Ligand showing an intense signal at *m/z*: 263.31 for $C_{17}H_{14}N_2O$, $[+H]^-$.

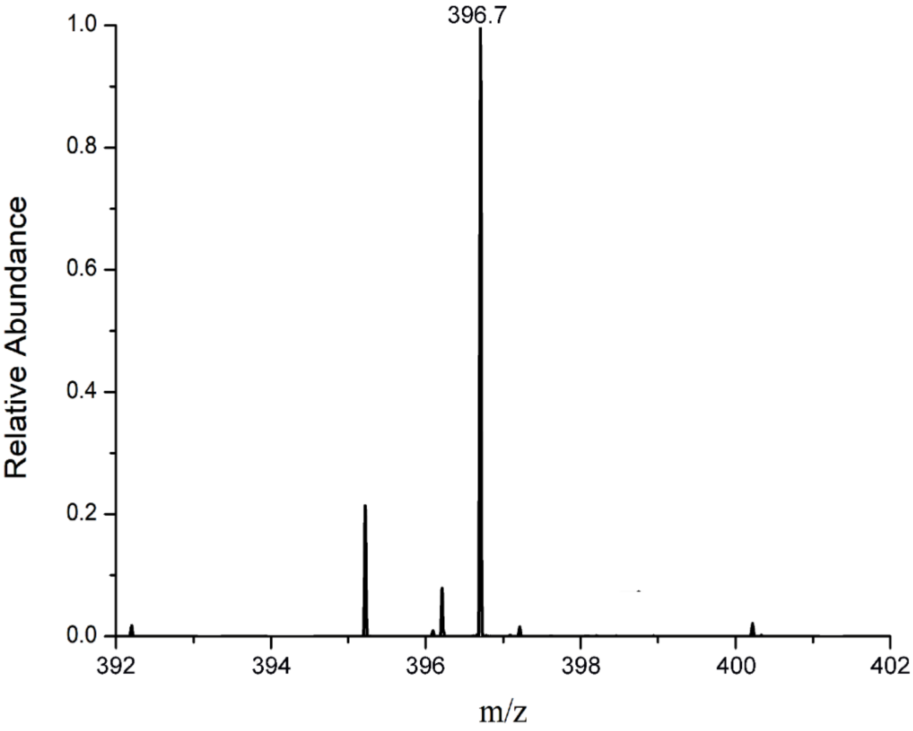

Figures S3. ESI-mass spectrum of the C1 showing an intense signal at *m/z*: 396.7 for $C_{22}H_{18}ClCuN_3O$ $[M+H]^+$.

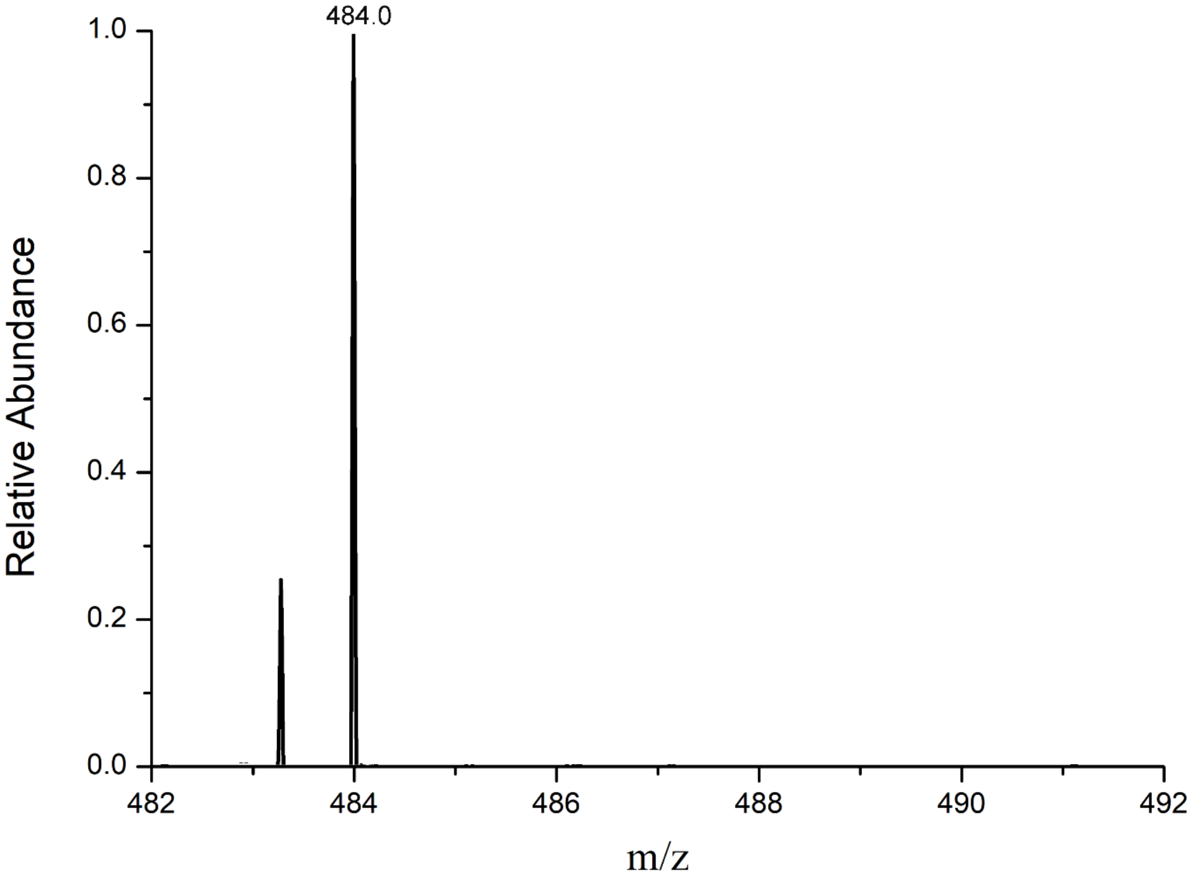

Figure S4. ESI-mass spectrum of the C2 showing an intense signal at *m/z*: 484.0 for $C_{22}H_{18}BrCuN_3O[M+H]^+$.

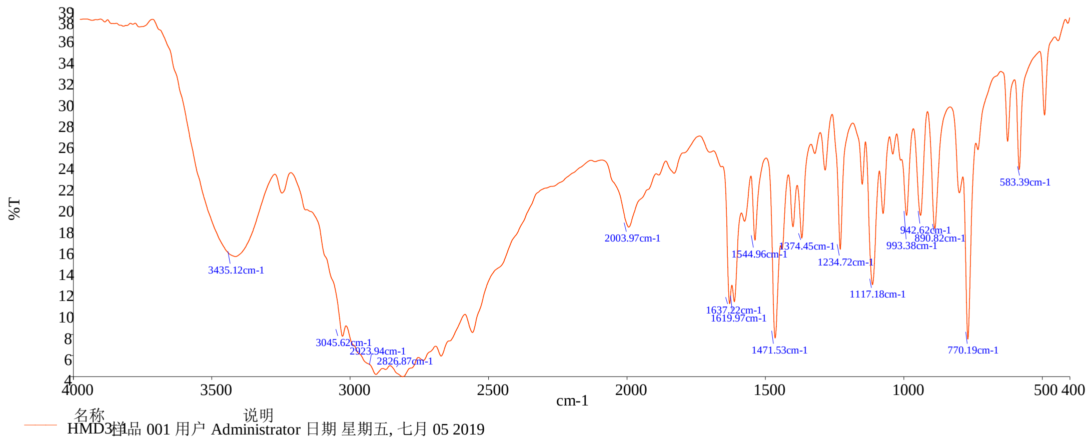

Figure S5. IR spectrum of the Ligand.

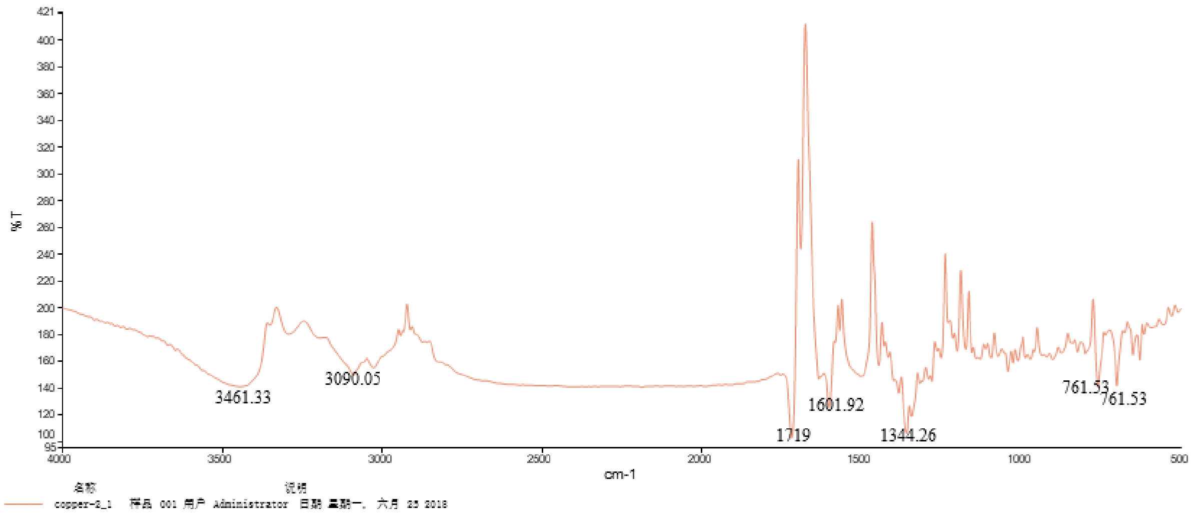

Figure S6. IR spectrum of Cu(II) complex (C1).

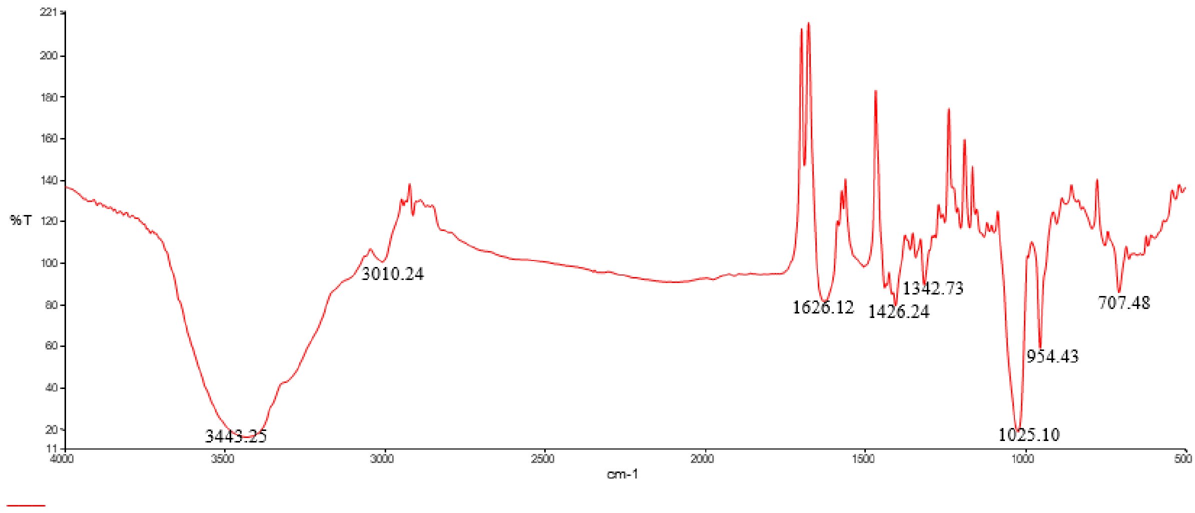

Figure S7. IR spectrum of Cu(II) complex (C2).
